# Thrombosis of the dorsal vein of the penis (Mondor’s Disease): A case report and review of the literature

**DOI:** 10.4103/0970-1591.70588

**Published:** 2010

**Authors:** Syed Sajjad Nazir, Muneer Khan

**Affiliations:** Department of Surgery and Urological Division Government Medical College, Srinagar, India

**Keywords:** Mondor’s Disease, penis, superficial, thrombophlebitis

## Abstract

Superficial thrombophlebitis of the dorsal vein of the penis (penile Mondor’s Disease) is an important clinical diagnosis that every family practitioner should be able to recognize. Dorsal vein thrombosis is a rare disease with pain and induration of the dorsal part of the penis. The possible causes comprise traumatism, neoplasms, excessive sexual activity, or abstinence. The differential diagnosis must be established with Sclerotizing lymphangitis and peyronies disease and doppler ultrasound is the imaging diagnostic technique of choice. Proper diagnosis and consequent reassurance can help to dissipate the anxiety typically experienced by the patients with this disease. We describe the symptoms, diagnosis, and treatment of the superficial thrombophlebitis of the dorsal vein of the penis.

## INTRODUCTION

Thrombosis of the superficial veins of the chest wall was first described by Mondor in 1939. It was Braun Falco, in 1958, who first described phlebitis of the dorsal veins of the penis within the context of generalized phlebitis.[1] In 1958, Helm published reports of isolated thrombosis of the superficial dorsal vein of the penis.[2] While Mondor’s Disease in the penis is rare, it is believed to be more common than is suggested by the mere 42 cases documented in literature.[3] The most extensive series corresponds to that of Finlay and Whiting.

Venous drainage of the penis begins at the base of the glans; a series of venous canals merge to form the dorsal vein of the penis, which in turn runs along a groove between the corpora and drains into the preprosthatic venous plexus. The circumflex veins orginate in the corpus spongiosa and extend around the corpus cavernosum on either side to merge with the deep dorsal vein perpendicularly. They are only present in the two distal thirds of the penis and total between 3 and 10 in number. Intermediate venules of the venous sinuses in turn form and drain into a capillary plexus beneath the tunica. This plexus system gives rise to emitting veins that generally extend obliquely between the layers of the tunica and drain into the circumflex vein at the dorsolateral level. The emitting veins in the proximal third of the penis merge over the dorsomedial surface of the corpus cavernosum bilaterally to form between 2 to 5 cavernous veins. In the hilum of the penis, these vessels pass between the pillars and the bulbar region receiving branches from each of them and joining with the internal pudendal veins.

This venous network can be affected by inflammatory processes under certain conditions, such as sexual trauma in the dorsal region and thrombophlebitis of the ventral portion.[2] We present a new case of superficial thrombophlebitis of the dorsal vein in the penis (Mondor’s Disease).

## CASE REPORT

An 18-year-old male presented with a history of painful dorsal induration on the proximal third of the penis for the previous week. The pain was throbbing and aching. There was no itching, discharge, hematuria, fever, dysuria, sexual dysfunction, or increased pain with an erection. He gave a history of recent masturbation without any trauma of any kind to the penis. He had not experienced this condition before and denied ever being infected with a sexually transmitted disease. He was taking no medications at the time of presentation. His family history yielded no helpful information. The patient reported that he abused neither alcohol nor smoke or took any other drugs.

A physical examination revealed a healthy young man in no apparent distress. A thin ropy cord was palpated superficially on his dorsal proximal penis. This cord included a dilated portion of approximately 0.6 cm in diameter. This indurated cord could be followed superiorly and extended into his pubic hair region by 2–3 cm. This cord was tender when palpated and the overlying skin was completely intact with no erythemia. An examination revealed no signs of lymphadenopathy in the groin region with no hernia.

The diagnosis indicated superficial thrombophlebitis of the dorsal vein of the penis. A doppler ultrasound revealed a dorsal induration corresponding to segmental thrombosis of the superficial dorsal vein of the penis - the rest of the trajectory remained permeable.

**Figure 1:**
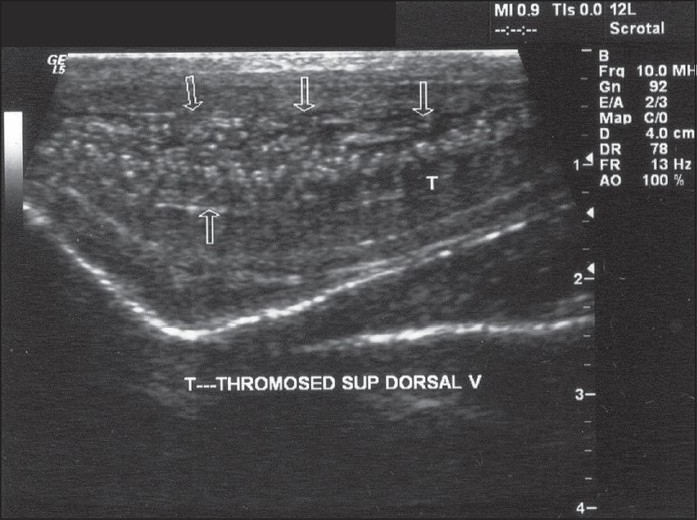
Ultrasound showing thrombosis of the superfi cial dorsal vein of the penis

[Figure 1] Ultrasound of the superficial vein of the penis demonstrating thrombosis

Supportive care was initiated, including a prescription for 50 mg of diclofenace twice daily, a 325 mg entericoated tablet of aspirin daily for anticoagulation, 200 mg of ofloxacin twice daily for prophylaxis, and heparin cream for local application for a period of 4 weeks as a result of which the pain subsided and induration disappeared. The patient was reassured of the benign nature of his condition and was instructed to refrain from any sexual experiences for this period.

## DISCUSSION

Thrombosis of the dorsal vein of the penis is a rare disorder that tends to affect males in the age range of 21–70 years old. Correctly diagnosing this benign condition is imperative so that the physician can allay the patient’s fears of having a sexually transmitted disease, erectile dysfunction, or cancer.

Many predisposing factors can lead to the development of thrombosis of the dorsal vein of the penis. These factors all relate back to Virchows traid of vessel wall damage, stasis, and a hypercoagulable state.[4] The reported causal factors comprise traumatisms, excessive sexual activity, prolonged sexual abstinence, local or distant infectious processes, venous obstruction secondary to bladder distension, pelvic tumors, disseminated pancreatic adenocarcinoma or constrictive elements used in certain sexual practices, and the abuse of certain intravenous drugs.[5,6]

Penile Mondor’s Disease can be diagnosed from medical history and a physical examination. The patient consistently presents with a rope-like cord on the dorsum of the penis. The cord is a thrombosed dorsal vein, which has become thickened and adherent to the overlying skin. Often, the lesion will extend superiorly into the suprapubic area. The vein may appear to be swollen and erythematous. The patient will report having a significant amount of pain, which can be either episodic or constant. In some cases, affected patients can also present irritative micturition syndrome.[7,8]

Superficial thrombophlebitis of the dorsal vein of the penis can be divided into three clinical stages: acute, subacute, and re-permeabilized. The acute stage tends to manifest in males in the range of 20 to 40 years old and typically manifests in the 24 hours following prolonged sexual activity, possibly secondary to vascular endothelial trauma.

Sclerosing lymphangitis and peyronies disease both need to be considered in the differential diagnosis of a painful, fibrotic lesion of the penis; however, Sclerosing lymphangitis is characterized by thickened and dilated lymphatic vessels whose morphology is serpiginous. Peyronies disease results from a thickening of the tunica albugenia and presents as a well-defined fibrotic plaque on the penis.

If doubt persists even after taking the medical history and performing the physical examination, consider ultrasonography. If the vein appears noncompressible, this is consistent with the diagnosis of venous thrombosis.[9]

Several methods of treating superficial thrombophlebitis of the dorsal vein of the penis have been proposed, none of which has been shown to significantly decrease duration. Anticoagulation with aspirin, heparin, or other antiplatelet agents will not expedite healing and is not necessary to prevent additional thrombosis. Currently, treatment is palliative for most patients. However, antibiotic therapy should be administered when cellulitis is suspected and vein stripping may be necessary for severe, persistent cases of Mondor’s Disease.[10] An injection of 0.5% bupivacaine hydrochloride subcutaneously in the region surrounding the affected vein has provided relief to patients who are in acute pain. Care should be taken to avoid injecting patients who have signs of infection, as this may exacerbate their condition. In the subacute and chronic stages, anti-inflammatory drugs and local heparin containing creams can be prescribed as was done in our case.[4]

Most cases resolve within 4 to 6 weeks, with re-permeabilization in 9 weeks. In persistent cases, surgery may prove necessary with a thrombectomy or resection of the superficial dorsal vein.

## References

[1] Bird V, Krasnokutsky S, Zhou H, Jarrahy R, Khan SA (1997). Traumatic thrombophlebitis of the superficial dorsal vein of the penis: an occupational hazard. Am J Emerg Med.

[2] Rodriguez-Faba O, Parra Muntaner L, Gomez Cisneros SC, Martin-Benito JL, Escaf-Barmadah S (2006). Thrombosis of the Dorsal vein of the Penis (Mondor’s phlebitis).A case report. Actas Urol Esp.

[3] Shapiro RS (1996). Superficial dorsal penile vein thrombosis (penile Mondors phlebitis):Ultra sound Diagnosis. J Clin Ultrasound.

[4] Griger DT, Angelo TE, Grisier DB (2001). Penile Mondor s disease in a 22- year old man. J Am Osteopath Assoc.

[5] Horn AS, Pecora A, Chiesa JC, Alloy A (1985). Penile thrombophlebitis as a persistent manifestation of pancreatic carcinoma. Am J Gastroenterol.

[6] Bennet RG, Leyden JJ, Decherd JW (1973). The heroin ulcer. New addition to the differential diagnosis of ulcers of the penis. Arch Dermatol.

[7] Sasso F, Gulino G, Basar M, Carbone A, Torricelli P, Alcini E (1996). Penile Mondors disease: an underestimated pathology. Br J Urol.

[8] Thomazeau H, Alno L, Lobel B (1983). Thrombosis of the dorsal vein of the penis. A propos of two cases. J Urol (Paris).

[9] Khan SA, Smith NL, Hu KN (1982). New perspectives in diagnosis and management of thrombophlebitis of the superficial dorsal vein of the penis. J Dermatol Surg Oncol.

[10] Swierzewski SJ, Denil J, Ohi DA (1993). The management of penile Mondor s phlebitis: superficial dorsal penile thromboses. J Urol.

